# Fecal microbiota profile in a group of myasthenia gravis patients

**DOI:** 10.1038/s41598-018-32700-y

**Published:** 2018-09-26

**Authors:** German Moris, Silvia Arboleya, Leonardo Mancabelli, Christian Milani, Marco Ventura, Clara G. de los Reyes-Gavilán, Miguel Gueimonde

**Affiliations:** 1 Neurology Service, Asturias Central University Hospital (HUCA), SESPA, Oviedo, Asturias Spain; 2 Department of Microbiology and Biochemistry of Dairy Products, Instituto de Productos Lácteos de Asturias (IPLA-CSIC), 33300 Villaviciosa, Asturias Spain; 3 Diet, Microbiota and Health Group, Instituto de Investigación Sanitaria del Principado de Asturias (ISPA), Oviedo, Spain; 4 Laboratory of Probiogenomics, Department of Chemistry, Life Sciences and Environmental Sustainability, University of Parma, Parma, Italy; 5 Microbiome Research Hub, University of Parma, Parma, Italy

## Abstract

The intestinal microbiota plays a key role in the maintenance of human health. Alterations in this microbiota have been described in several autoimmune diseases, including nervous system diseases. Nevertheless, the information regarding neuromuscular conditions is still limited. In this study, we aimed at characterizing the intestinal microbiota composition in myasthenia gravis patients (MG). To this end fecal samples were taken from ten patients, with antibodies against the acetylcholine receptor, and ten age and sex matched controls from the same population (Asturias region, Spain). Fecal samples were submitted to microbiota analyses by 16S rRNA gene profiling, bifidobacterial ITS-region profiling and qPCR. The fecal levels of short chain fatty acids were determined by gas chromatography. MG patients were found to harbor lower relative proportions of *Verrucomicrobiaceae* and *Bifidobacteriaceae*, among others, and increased of the phylum Bacteroidetes and the family *Desulfovibrionaceae*. The increase of these latter microbial groups was also confirmed at quantitative level by qPCR. In contrast, no statistically significant differences were found between MG patients and the control group in the bifidobacterial population at the species level or in short chain fatty acids profiles. Our data indicates an altered fecal microbiota pattern in MG patients and point out at specific microbiota targets for intervention in this population.

## Introduction

The human gastrointestinal tract (GIT) harbours a very complex and dynamic microbial community, the so called *gastrointestinal microbiota*. This complex microbial ecosystem exceeds the number of host cells^1^. It contains a gene set 100 times larger than that of the human genome, carrying out many functions that are not encoded in our own genome^2^. The bacterial colonization of the human gut with this microbiota plays an essential role for the development and maintenance of an appropriate metabolic and immune homeostasis in the host^3,4^.

An increasing body of scientific evidence has arisen during the last years indicating that the microbiota-host interaction affects not just the gut environment but also distal organs^5–7^. Among these, several studies strongly suggest that the intestinal microbiota may interplay with the nervous system and the brain^8^. Animal studies have evidenced the potential of the gut microbiota to modulate pain perception^9–11^, behaviour, mood and stress response^5,12,13^. The gut microbiota is able to produce neuroactive molecules such as histamine, acetylcholine or GABA, among others^14^. Actually, the gut is the second organ with more nerve cells in our body, behind the brain, and it has its own nervous system, the Enteric Nervous System (ENS), which has led to the concept of the *Gut Brain*^15^. However, the role of the microbiota in this context, especially regarding the neuro-muscular diseases, is only barely known. Several studies have focused on autoimmune diseases such as inflammatory bowel disease (IBD), rheumatoid arthritis or multiple sclerosis but, to date, there are no data available on other pathologies such as myasthenia gravis (MG)^16^.

MG is an autoimmune disorder caused by antibodies directed against the postsynaptic muscle membrane. This process leads to focal or generalized muscle weakness and fatigability^17^. Ocular muscle weakness is the most common presenting symptom, but often symptoms extend to bulbar, limb, axial, and ventilator muscles, resulting in generalized MG. Autoantibodies against the acetylcholine receptor (AChR), muscle-specific kinase (MUSK), and lipoprotein-related protein 4 (LRP4) are well established in MG patients; anti AChR antibodies can be detected with routine assays in the 70–80% of all patients with MG^17^. The MG with anti AChR antibodies (AChR-MG) is classified as early-onset (onset of their first symptom before the age of 50 years) or late-onset (first symptom after the age 50 of years)^18^.

Pyridostigmine, the most commonly used acetylcholinesterase inhibitor, provides symptomatic therapy in MG patients, but its use alone is insufficient to control symptoms in mild symptomatic patients. Corticosteroids are considered the first-line immunosuppressive therapy but some steroid-sparing immunosuppressive agents, such as azathioprine, mycophenolate mofetil or tacrolimus, are also used. Plasma exchange and intravenous immunoglobulin are started if a MG crisis is suspected^19^.

While the precise etiology of MG remains obscure, it is likely that the development of this disease is dependent upon environmental factors in genetic predisposed patients. The commensal bacteria that colonize the gastrointestinal tract may also play a role in the development of the MG; the confirmation of this hypothesis would support new therapeutic strategies^16^.

In this context we aimed at characterizing the intestinal microbiota composition in seropositive myasthenia gravis patients with anti-AChR antibodies in comparison with a control group of age and sex matched subjects.

## Results

Sequencing of the PCR products obtained by amplification of the V3 region of the 16S rRNA gene produced an average of ~ 65,000 filtered partial sequences per sample, with an approximate length of 178 bp. The rarefaction curve obtained using the Shannon index shows that the plateau phase was reached, indicating enough sequencing depth (Supplementary Fig. S1).

**Figure 1:**
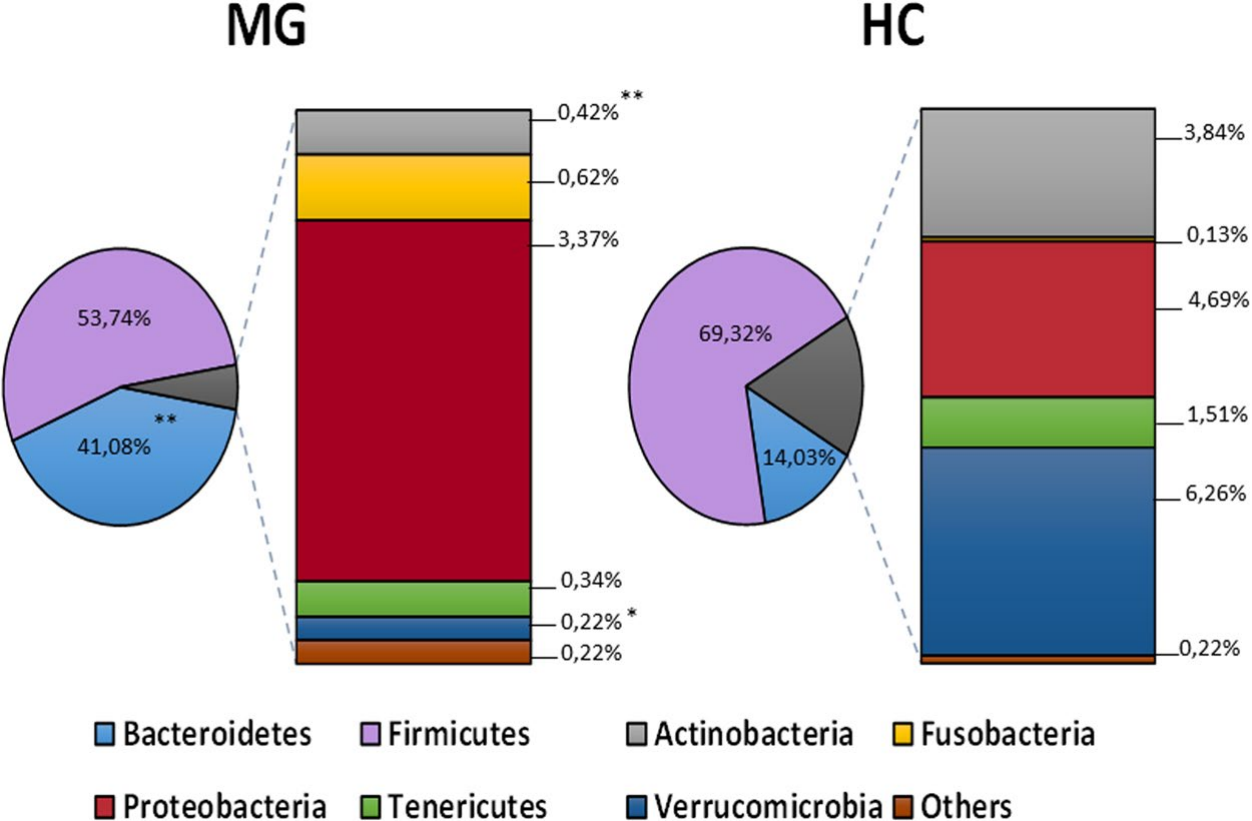
Aggregate microbiota composition in faecal samples from myasthenia gravis group (MG) and healthy control group (HC) at the phylum level. Relative proportions (%) are shown. In the comparison between MG and HC groups * indicates p < 0.05, and ** indicates p < 0.01.

**Figure 2:**
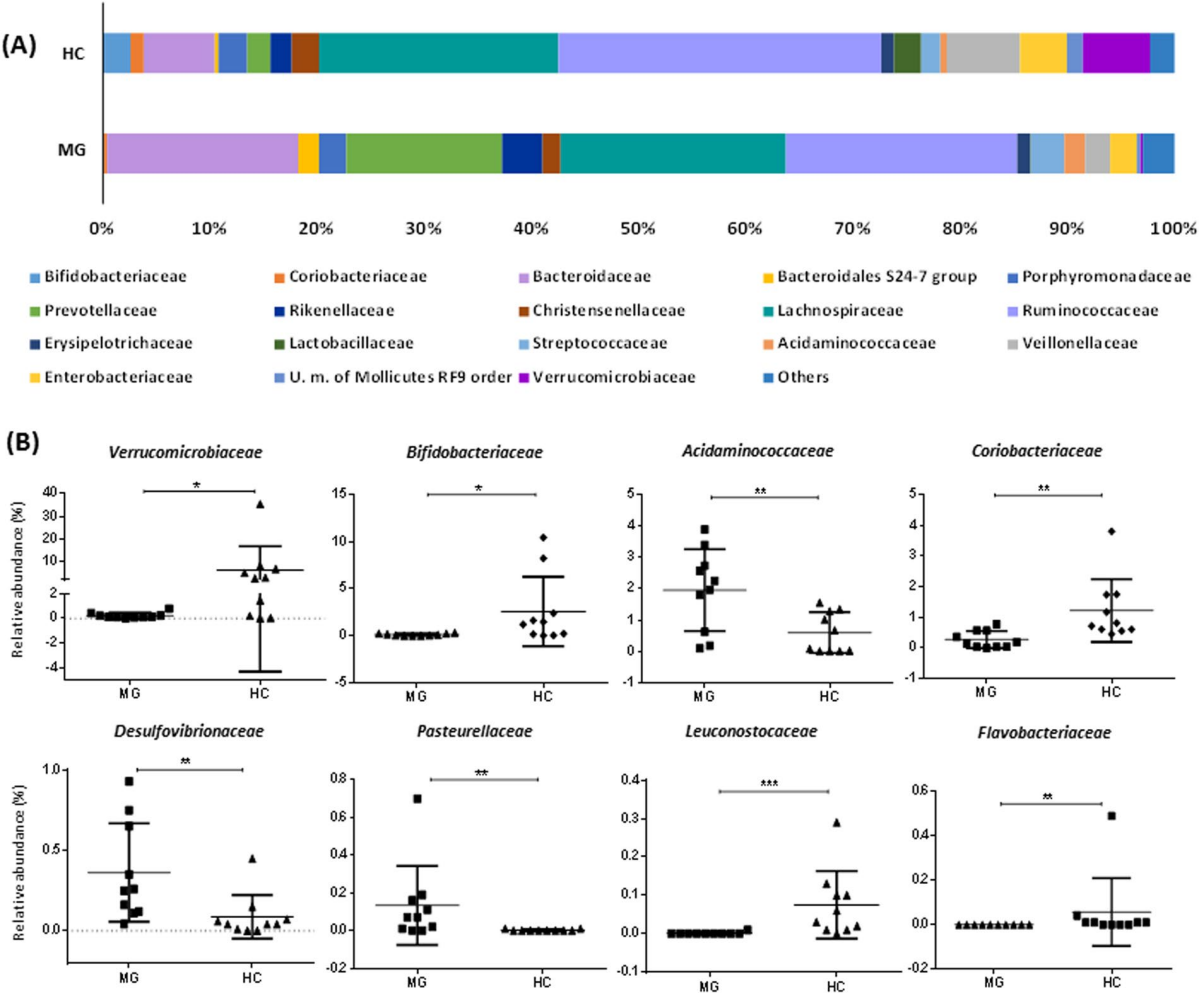
Aggregate microbiota composition in faecal samples from myasthenia gravis group (MG) and healthy control group (HC) at the family level (**A**). Dot plots (values, mean and SD) of the families significantly different between the two groups of study are shown (**B**). * indicates p < 0.05, ** indicates p < 0.01, *** indicates p < 0.001.

The analyses of the 16S rRNA gene profiling of fecal samples evidenced that both groups of volunteers, MG and control, differed in their beta-diversity (Supplementary Fig. S2) but did not show differences in the alpha-diversity (data not shown). When assessing the microbiota composition at the phylum level we found that Firmicutes were the dominant fecal microbes in both groups of individuals (Fig. 1). However, statistically significant differences (p < 0.05) were observed for some of the subdominant phyla between both groups, with MG patients showing an increased relative proportion of Bacteroidetes and reduced proportions of Actinobacteria and Verrucomicrobia. When the 16S rRNA gene profiling data were analyzed at family level (Fig. 2), MG patients were found to harbor significantly lower (p < 0.05) relative proportions of the families *Verrucomicrobiaceae* and *Bifidobacteriaceae* as well as *Coriobacteriaceae*, *Leuconostocacceae* and *Flavobacteriaceae*. On the contrary MG patients harbored higher proportions (p < 0.05) of *Acidaminococcaceae*, *Desulfovibrionaceae* and *Pasteurellaceae*.

**Figure 3:**
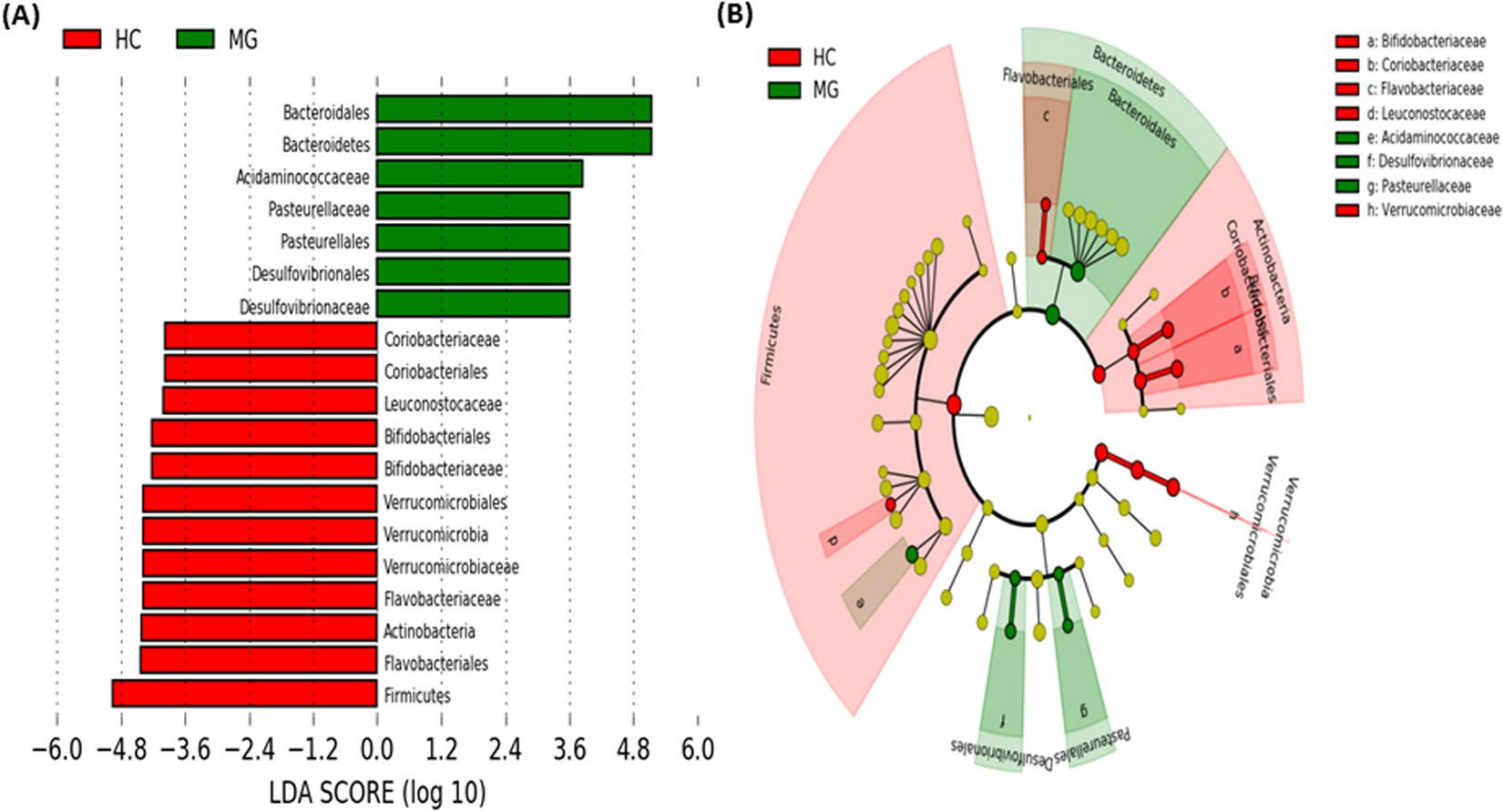
Linear discriminant analysis (LDA) scores of taxa significantly altered in myasthenia gravis group are shown in (**A**) (LDA scores > 2 and significance of *p* < 0·05 as determined by Wilcoxon’s signed-rank test). The most differentially abundant taxa in (red) healthy control (HC) and (green) myasthenia gravis group are represented as a cladogram in (**B**) that was generated from LDA effect size analysis data in (**A**). The color intensity of each dot is proportional to its effect size (**B**).

Linear discriminant analysis effect size (LEFSe) was performed by using the 16S rRNA gene profiling data in order to identify the phylotypes responsible for the differences between MG and healthy controls (Fig. 3). The results identified increased abundance of Bacteroidetes or *Desulfovibrionaceae*, among others, in MG patients. *Bifidobacteriacea*e, *Verrucomicrobiaceae*, *Leuconostococcaceae*, *Flavobacteriaceae* and *Coriobacteriaceae* were the most differentially abundant taxa in the healthy control group.

The determination of the absolute levels of selected microbial groups by qPCR (Table 1) showed significantly (p < 0.05) higher counts of total bacteria, and *Desulfovibrio* and *Bacteroides*-group in MG patients. However, statistically significant differences between groups were not found for the levels of the other microorganisms analyzed, although a trend (p = 0.052) towards reduced counts of *Akkermansia* was evidenced in MG patients.

**Table 1:** Levels (Log n° cells /gram of feces) of different intestinal microbial groups determined by quantitative PCR in myasthenia gravis group (MG) and healthy control group (HC). Median and interquartile range values are represented.

Microbial Group	MG	HC	*p*. *value*
*Akkermansia*	7.44 (6.49–7.76)	7.97 (6.55–8.66)	0.052
*Bacteroides* group	10.57 (10.30–11.03)	9.54 (8.69–10.24)	**0.002**
*Bifidobacterium*	8.84 (8.61–9.25)	9.16 (8.75–9.60)	0.481
*Clostridia cluster XIVa* group	9.05 (8.51–9.50)	9.05 (8.39–9.34)	0.529
*Desulfovibrio*	8.64 (6.56–9.08)	6.32 (5.67–7.23)	**0.015**
*Enterobacteriaceae*	8.97 (8.20–9.57)	8.51 (6.96–9.05)	0.123
*Faecalibacterium prausnitzii*	8.22 (7.91–8.51)	7.87 (7.61–8.17)	0.089
Total bacteria	11.28 (11.02–11.74)	10.83 (10.47–11.06)	**0.009**

**Figure 4:**
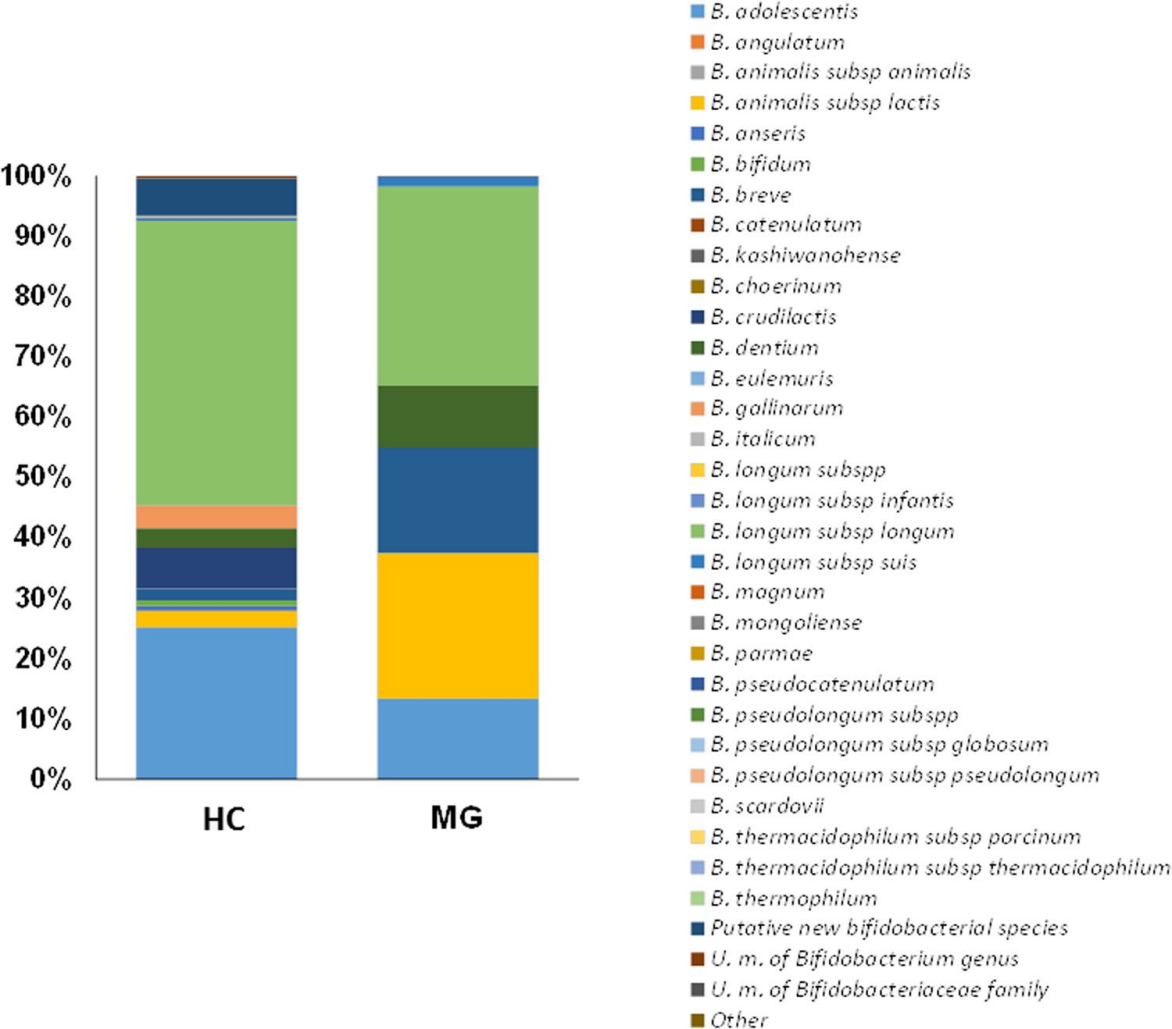
Aggregate bifidobacterial microbiota composition in faecal samples from myasthenia gravis group (MG) and healthy control group (HC) at the species level. Relative proportions (%) are shown.

A large inter-individual variability was observed for the bifidobacterial population as assessed by ITS profiling. This high variability likely determined the lack of statistically significant (p < 0.05) differences among both volunteers’ groups. Nevertheless, some differences between the groups become apparent, with the bifidobacterial population on healthy controls dominated by *Bifidobacterium longum* subsp. *longum* followed by *Bifidobacterium adolescentis*. However, MG patients showed high relative proportions of *Bifidobacterium animalis* subsp. *lactis*, *Bifidobacterium breve* and *Bifidobacterium dentium* (Fig. 4).

Regarding the short chain fatty acids (SCFA), no statistically significant differences were observed between the healthy control and the MG groups. The levels of the main SCFA were comparable between groups, with acetate being present at a concentration (median [IQR]) of 54.7 [39.9–56.6] and 61.1 [45.0–75.6] mM in the MG and control groups, respectively. Propionate concentration was 15.5 [10.0–27.5] and 19.8 [14.2–22.0] mM, butyrate 10.7 [5.5–23.2] and 16.3 [11.2–24.4] mM, iso-butyrate 2.3 [1.8–3.3] and 2.8 [2.4–3.3], iso-valerate 2.8 [2.4–4.7] and 4.5 [3.8–5.4] and valerate 2.6 [1.7–4.0] and 3.2 [2.6–3.8] mM, for the MG and control groups, respectively.

## Discussion

To the best of our knowledge this is the first study assessing the intestinal microbiota composition in AChR-MG patients as compared with matched healthy controls. Our results indicate a severe dysbiosis in the gut microbiota of these patients. This is in line with the recent evidence indicating the association of intestinal microbiota aberrancies and different autoimmune diseases such as allergy^20,21^, Type-1 diabetes^22^, inflammatory bowel disease^23,24^, lupus erythematosus^25^, multiple sclerosis^26,27^, rheumatoid arthritis^28,29^ or spondyloarthritis^29^, among others. However, there is not a common microbial dysbiosis pattern associated with these different autoimmune conditions and, therefore, the microbiota alterations found in MG patients do not seem to be extrapolated to other autoimmune conditions.

A reduced microbial diversity has been reported in some autoimmune diseases, such as rheumatoid arthritis^28,29^, Crohn’s disease^24^ or active multiple sclerosis^26^. However, in agreement with our data for MG, other authors did not observe such reduction of the microbiota diversity in multiple sclerosis^27^, systemic lupus erythematosus^25^ or type-1 diabetes^22^. Some studies have linked the decrease in alpha-diversity to the disease duration and/or activity^24,28^. This may partially explain why in our case differences in alpha-diversity were not found, since nine out of our ten MG patients have been diagnosed during the previous year, and the effect on bacterial diversity may require longer courses of disease.

In general, in control volunteers the levels of the different bacterial groups analyzed by qPCR were within the range of those previously found by us using the same technique, in subjects of similar age from our region^30^. Similarly, the 16S rRNA gene profiling data are also in agreement with previous observations by other authors in other geographical locations^31^. In the case of our MG patients the intestinal microbiota profile at the phylum level showed reduced proportions of Verrucomicrobia and Actinobacteria but increased proportions of Bacteroidetes, and these results were corroborated by qPCR data. Moreover, LEFSe analysis allowed identifying the differentially abundant taxa between both volunteers’ groups. Consistent with the previously commented results, the analyses pointed out at the Bacteroidetes and *Desulfovibrionaceae*, together with *Pastereullaceae*, as the increased taxa in MG patients. The taxa showing higher scores in the LEFSe analyses in the healthy control subjects included, among others, the families *Bifidobacteriaceae*, *Verrucomicrobiaceae* and the phylum Firmicutes. These results underline the clearly different microbiota profiles in MG versus healthy control subjects.

In contrast to the results obtained on microbial composition, no differences between MG and control groups were observed for the fecal concentration of SCFA. Similarly, the relative proportions of the different species of the genus *Bifidobacterium* did not reach significant difference between groups. This was likely due to the large inter-individual variability found, and the limited sample size, since the bifidobacterial profiles at the species level were apparently different between MG and healthy control subjects.

It is important to point out that the patients’ therapy may have an effect in the microbiota, which may account for some of the differences observed in the present study and, in general, also when studying other different autoimmune diseases. To this regard, as in our case, a cross-sectional study design is often used for comparing patients under treatment with matched healthy controls. However, this design does not allow overruling the impact of medication. The comparison with recently diagnosed patients, before medical treatment, may provide a more precise picture of the disease-specific microbiota alterations. Moreover, in this study we focused on a specific subset of MG patients, including only AChR-MG cases. However, the encouraging results obtained support new studies focusing also on other MG patient subgroups with different clinical presentation and biomarkers. Our results, in agreement with the hypotheses of other authors^16^, suggest the potential interest of therapies aimed at modulating the gut microbiota in the management of MG.

## Conclusion

This study report, by the first time, the altered fecal microbiota pattern displayed by AChR-MG patients in comparison with age and sex matched healthy controls. Our results point out at specific microbiota targets for the development of probiotics, prebiotics or other microbiota-modulating tools for MG patients.

## Material and Methods

### Volunteers and fecal samples collection

Fecal samples from twenty volunteers, ten of them suffering AChR-MG (nine late-onset and one early-onset) (Table 2) and ten sex and age matched controls were obtained at the Asturias Central University Hospital (HUCA, Asturias, Spain) during the period of June-August 2017. The AChR-MG patients included seven women (73.3 ± 9.7 years-old) and 3 males (65.3 ± 5.8 years-old) and an identical number of women (72.6 ± 9.9 years-old) and men (66.0 ± 8.6 years-old) were included in the control group. None of the subjects had suffered any abdominal chirurgic intervention or consumed antibiotics, probiotics or anti-acids during the previous two months or reported gastrointestinal symptoms during the previous year. The study was approved by the Regional Ethical Committee of Asturias Public Health Service (SESPA) and an informed written consent was obtained from each adult volunteer. All experiments were carried out in accordance with the Declaration of Helsinki on Ethical Principles for Medical Research Involving Human Subjects and with approved guidelines and regulations.

**Table 2:** Characteristics of Myasthenia Gravis patients included in this study.

Clinical Characteristics					AChRA** Concentration (nmol/L)	Therapy						
	Age	Symptoms duration (months)	Gender	MGFA* Clinical Classification		Pyridostigmine		Steroid		Immuno-suppressives type, months from therapy onset, doses (mg/day)	Intravenous Immunoglobulin (courses)	Thymectomy
						Months from therapy onset	Dose (mg/day)	Months from therapy onset	Dose*** (mg/day)			
1	62	10	Male	IIb	1.07	10	240	NO		NO	NO	NO
2	77	25	Female	IIIa	2.48	1	180	NO		NO	NO	NO
3	69	360	Female	I	4.09	NO		NO		NO	NO	NO
4	66	84	Female	II	1.81	NO		NO		NO	NO	NO
5	62	18	Male	IIb	37,356	13	180	NO		NO	YES (2)	NO
6	78	29	Female	I	0.66	28	180	28	12.5	Mycophenolate mofetil, 24, 1500	NO	NO
7	84	24	Female	IIIb	9.84	13	90	12	30	Azathioprine, 12, 100	YES (1)	NO
8	82	7	Female	IIb	1.04	5	180	5	15	NO	YES (2)	NO
9	57	15	Female	IIb	238,58	16	300	16	10	Tacrolimus, 2, 1****	YES (5)	NO
10	72	5	Male	I	20.42	NO		NO		NO	YES (1)	NO

^*^MGFA: Myasthenia Gravis American Foundation Clinical Classification.

**AChRA: acetylcholine receptor antibodies.

***In dose equivalent prednisone.

****Previously patient was on azathioprine, 9 months, 150 mg/day and mycophenolate mofetil, 6 months, 2000 mg/day, drugs withdrawn due to inefficacy.

The volunteers provided a fresh fecal sample that was immediately frozen (−20 °C) until analyses. For analyses the fecal samples were melted, diluted 1/10 in PBS solution and homogenized in a LabBlender 400 stomacher at full speed for 3 mins. Then DNA was extracted from 1 ml of the fecal homogenate by using the QIAamp DNA stool kit (Qiagen, GmbH, Hilden, Germany) as previously described^32^ and was stored at −20 °C until use.

### Analyses of intestinal microbiota

#### Analysis of fecal microbial groups by 16S rRNA gene profiling and bifidobacterial ITS profiling

The extracted DNA was used for the assessment of the microbial populations by 16S rRNA Gene Sequence-based microbiota analysis. In brief, partial 16S rRNA gene sequences were PCR-amplified using previously described primers^33^ and the amplicons were sequenced in a MiSeq (Illumina) platform (GenProbio srl, Parma, Italy). The individual sequence reads obtained were filtered, trimmed and processed as described by Nogacka and coworkers^34^. 16S rRNA Operational Taxonomic Units (OTUs) were defined at ≥97% sequence homology using uclust^35^. All reads were classified to the lowest possible taxonomic rank using QIIME and a reference dataset from the SILVA database^36^.

To gain further insight into the fecal bifidobacterial populations a profiling of known bifidobacterial species was performed using the primer pair Probio_bif_uni/Probio_bif_rev, an improved bifidobacterial ITS database encompassing all publicly available bifidobacterial genomes and a custom bioinformatics script, as described previously^37^.

#### Analysis of fecal microbial groups by quantitative PCR

The levels of Bacteroides-Prevotella-Porphyromonas group, Faecalibacterium, Bifidobacterium, Lactobacillus-group, Staphylococcus, Akkermansia, Enterobacteria, Clostridium XIVa group, as well as of total bacteria, were determined by quantitative PCR (qPCR) using previously described primers and conditions^38,39^.

#### Determination of SCFA levels in feces

The analysis of SCFA was performed by gas chromatography in order to determine the concentrations of acetate, propionate, isobutyrate, butyrate, isovalerate and valerate. Cell free-supernatants (100 μl) from fecal homogenates, prepared as indicated formerly, were mixed with 450 μl methanol, 50 μl internal standard solution (2-ethylbutyric 1.05 mg/ml), and 50 μl 20% v/v formic acid. This mixture was centrifuged and the supernatant obtained was used for quantification of SCFA by GC in a system composed of a 6890NGC injection module (Agilent Technologies Inc., Palo Alto, Ca, USA) connected to a flame injection detector (FID) and a mass spectrometry (MS) 5973N detector (Agilent), as described elsewhere^40^.

### Statistical analysis

Some of the variables analyzed were not normally distributed and/or lacked homogeneous variances; therefore the non-parametric U-Mann-Whitney test was used for comparing the different population groups with the control group. Statistical analyses were conducted using the IBM SPSS Statistics for Window Version 23.0 (IBM Corp., Armonk NY) software. Linear discriminant analysis (LDA) effect size (LEfSe) was used to estimate the taxa of microorganisms differing significantly between myasthenia patients and control individuals. LEfSe uses Kruskal-Wallis sum-rank test (with an alpha significance level of 0.05) to detect features with significantly different abundances, followed by a logarithmic linear discriminant analysis (LDA) (with an effect-size threshold of 2.0) to estimate the effect-size of each differentially abundant feature^41^.

### Nucleotide sequence accession numbers

The raw sequences reported in this article have been deposited in the NCBI Short Read Archive (SRA) with the accession number PRJNA450610.

## Electronic supplementary material

Supplementary file
