# Supplementary material for: Fecal microbiota profile in a group of myasthenia gravis patients
Source: Sci Rep. 2018 Sep 26;8:14384. doi: 10.1038/s41598-018-32700-y (PMC6158187; doi:10.1038/s41598-018-32700-y)

Supplementary file

Fecal microbiota profile in a group of myasthenia gravis patients

Authors: German Moris[1], Silvia Arboleya[2,3], Leonardo Mancabelli[4], Christian Milani[4], MarcoVentura[4,5], Clara G. de los Reyes-Gavilán[2,3], Miguel Gueimonde[2,3*]

[1] Neurology Service, Asturias Central University Hospital (HUCA), SESPA, Oviedo, Asturias, Spain.

[2] Department of Microbiology and Biochemistry of Dairy Products, Instituto de Productos Lácteos de Asturias (IPLA-CSIC), 33300 Villaviciosa, Asturias, Spain.

[3] Diet, Microbiota and Health Group, Instituto de Investigación Sanitaria del Principado de Asturias (ISPA).

[4] Laboratory of Probiogenomics, Department of Chemistry, Life Sciences and Environmental Sustainability, University of Parma, Italy.

[5] Microbiome Research Hub, University of Parma, Italy.

Corresponding author: Miguel Gueimonde. IPLA-CSIC. Ctra. Infiesto s/n, 33300 Villaviciosa, Asturias, Spain. Tel. +34 985892131, Fax. +34 985892233. E-mail;.e-mail: mgueimonde@ipla.csic.es

Supplementary figures

Figure S1. Rarefaction curves generated for the 16S rRNA sequences obtained from the fecal samples by using the Shannon index. C1 to C10: samples from individuals of the control group. MG1 to MG10: samples from individuals of MG group.

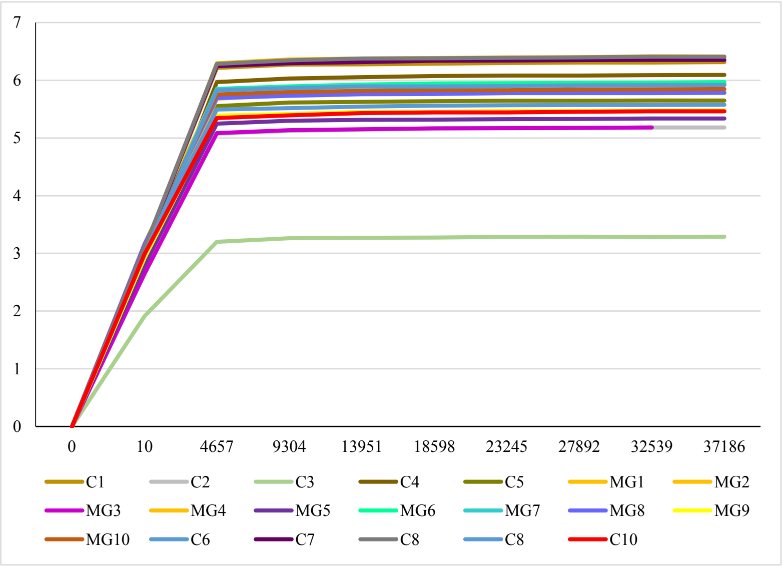

Figure S2. PCoA obtained with the beta-diversity values from the myastenia gravis patients (green) and the age and sex matched control group (red).

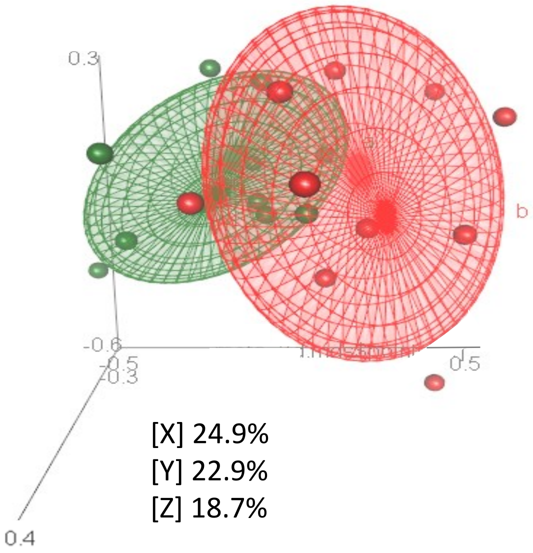

Supplement: Supplementary file 1 — Supplementary file [file 41598_2018_32700_MOESM1_ESM.pdf]
